# Teledermatology and Virtual Visits for Acne Management: A Review

**DOI:** 10.1177/12034754241291028

**Published:** 2024-11-01

**Authors:** Shanti Mehta, Dea Metko, Mahan Maazi, Ou Jia (Emilie) Wang, Monica K. Li

**Affiliations:** 1 Temerty Facuty of Medicine, University of Toronto, Toronto, ON, Canada; 2 Michael G. DeGroote School of Medicine, McMaster University, Hamilton, ON, Canada; 3 Faculty of Medicine, University of British Columbia, Vancouver, BC, Canada; 4 Department of Dermatology & Skin Science, Faculty of Medicine, University of British Columbia, Vancouver, BC, Canada; 5 Vancouver Skin MD, Vancouver, BC, Canada

**Keywords:** acne, acne vulgaris, teledermatology, telemedicine, virtual

## Abstract

Acne vulgaris is a common dermatological condition requiring individualized management. Teledermatology provides convenience and accessibility that is highly suitable for this dermatological condition. Herein, our review aimed to describe the current state of teledermatology in the context of acne management and to assess patient satisfaction, adherence to virtual care, and the experiences of health care providers in delivering remote dermatological services. A systematic search for articles was conducted in Medline, Embase, and PubMed according to the Preferred Reporting Items for Systematic Reviews and Meta-Analyses Extension for Scoping Reviews. Title, abstract, full-text screening, and data abstraction were carried out in duplicate. One thousand one hundred three nonduplicate articles were screened based on title and abstract review. A total of 21 studies were included in the review. It was found that teledermatology is well-suited to the effective management of acne vulgaris. When compared to in-person care, teledermatology resulted in similar outcomes on several acne-grading scales. Additionally, patient satisfaction was comparable, with a large proportion of patients preferring virtual care to traditional in-person visits. However, compliance was found to be lower for virtual care. There are several secondary benefits to telemedicine, including time savings and greater accessibility to care for rural patients. Teledermatology is an evolving, promising medium for acne management for both clinicians and patients. Future research comparing the effectiveness of different teledermatology platforms, its limitations and pitfalls, and integration of patient and physician preferences to improve treatment outcomes is warranted.

## Introduction

The advent of telemedicine, accelerated by the COVID-19 pandemic, has revolutionized the management of medical conditions, including dermatology.^1-6^ High-resolution imaging, digital health applications, and telemonitoring tools have all played a pivotal role in enhancing diagnostic accuracy, treatment monitoring, and overall patient engagement in virtual care settings.^7-11^ Teledermatology, a form of telemedicine in the field of dermatology, has become an increasingly popular approach to receiving care for dermatological conditions, especially for patients affected by acne.^12-16^

Acne vulgaris is a highly common dermatological condition characterized by comedones, papules, pustules, and nodules, which appear on the face, back, and chest and can lead to scarring.^17-19^ Acne can cause significant psychosocial implications including a decreased quality of life, self-esteem, and mental well-being and is emotionally distressing for patients particularly those with severe presentations.^20-25^ Acne also affects individuals of various ages, ethnicities, and genders.^26-29^ Recognizing the need for innovative solutions to address acne’s unique challenges, teledermatology has increasing appeal for patients to access specialized care remotely. Existing literature reflects a growing body of evidence supporting the use of teledermatology in acne management; however, there is value in summarizing learnings and current challenges to improve the medium for future applications.

Our review aimed to assess the current state of teledermatology in the context of acne management and to assess patient satisfaction, adherence to virtual care, and the experiences of health care providers in delivering remote dermatological services. Herein, we aimed to provide an increased understanding of how teledermatology can contribute to acne care for patients while aligning it with patient values and sustainable health care delivery models.

## Methods

We conducted a comprehensive literature search using Medline (since 1946), Embase (since 1974), and PubMed up to November 21, 2023, through the OVID interface, adhering to Preferred Reporting Items for Systematic Reviews and Meta-Analyses Extension for Scoping Reviews guidelines using the search strategy outlined in Supplemental Appendix 1.

We selected peer-reviewed, English-language studies focusing on acne vulgaris management via teledermatology. At the full-text screening stage, studies not meeting our pre-defined PICOS (Population, Intervention, Comparison, Outcomes, Study Design) criteria were excluded. Two authors (D.M. and S.M.) independently screened titles, abstracts, and full texts, resolving discrepancies through discussion. Both authors resolved any conflicts in this screening process upon discussion with the other authors. We also conducted citation chaining to ensure a comprehensive coverage.

Data extraction, carried out in duplicate, involved aggregating key study details into an external Excel sheet. These included the following: “Title,” “Authors,” “Published Year,” “DOI,” “Study Type,” “Study Objective,” “Total patients,” “Mean age (yrs),” “Age range (yrs),” “Sex,” “Type of acne,” “Location of acne,” “Duration of acne,” “Acne scale used,” “Treatment for acne,” “Type of treatment,” “Number of treatment sessions,” “Duration of treatment,” “Teledermatology performed,” “Type of teledermatology platform,” “ Total number of teledermatology sessions for each patient,” “Mean number of teledermatology sessions,” “Comparison of teledermatology vs in-person,” “Did the study use a patient and/or physician satisfaction questionnaire?,” “What questionnaire(s) did they use?,” “Overall results of questionnaire,” “ Efficacy of teledermatology for acne,” “Challenges with teledermatology for acne,” “Any secondary outcomes,” and “Summary of study.”

After data collection, we determined that quantitative evidence synthesis was not feasible due to differences between design and measures of the studies included in our review. As such, our results are presented in narrative form for each outcome.

## Results

Our literature search yielded 1103 nonduplicate articles, of which 1014 were excluded based on title and abstract review (Supplemental Figure S1). No studies were excluded based on language. A total of 21 studies were ultimately included in the review, 2 of which were conference abstracts. The 21 included publications were published between 2009 and 2023, which include 1 case-control study, 1 cohort study, 9 cross-sectional studies, 1 observational prospective study, 1 prospective longitudinal study, 4 randomized controlled trials, 2 reliability pilot studies, 1 retrospective study, and 1 survey (Supplemental Table S1).

A total of 7273 patients were included across all studies. Several different scales were used to measure acne quality and characteristics including the Investigator’s Global Assessment (IGA), Patient Health Questionnaire 9, International Consensus Conference on Acne Classification System, Dermatology Life Quality Index, Global Acne Grading System, Global Acne Severity Scale, Total Lesion Counting Scale, Network Oriented Research Assistant, Total Inflammatory Lesion Counts, Frontal Inflammatory Lesion Counts and Leeds. Isotretinoin was specified as a treatment for acne in 10 of the studies; however, the use of spironolactone and topical antibiotics was also described. Mobile phone photographs were the most common type of teledermatology platform used, in addition to video calls, web portals, and mobile apps. Finally, 9 of the 21 studies compared virtual teledermatology to in-person care.

## Discussion

### Effectiveness of Teledermatology for Acne Management

Evaluation of 21 studies yielded valuable insight into the application of teledermatology in the management of acne vulgaris. Findings consistently reported that teledermatology is well-suited to the effective management of acne vulgaris. Two studies assessed the feasibility of implementing teledermatology practices, specifically for patients with moderate-to-severe acne for which isotretinoin treatment is recommended. In both instances, it was found that utilizing teledermatology, via the acquisition of photographs on a mobile phone, adequately met the needs of patients.^30,31^ The Global Acne Grading System (GAGS) was used in numerous studies, with 1 revealing that an 87.94% reduction in GAGS score was experienced by patients using teledermatology for acne management.^31^ This highlights the efficacy of teledermatology in reducing acne scores and resulting in positive patient outcomes.

Isotretinoin can be obtained through a prescription from a physician and may require a consultation by a dermatologist. The dosage of isotretinoin can be increased to amplify the therapeutic effect or decreased to mitigate side effects. The adjustment of isotretinoin dosing has typically been carried out in-person; however, a study on the feasibility of teledermatology found that dermatologists were comfortable remotely adjusting the dosing of isotretinoin without a synchronous in-person patient meeting.^32^

### Comparative Analysis with Traditional In-Person Dermatology Consultations

To precisely measure the accuracy of teledermatology for acne management, it is imperative to compare teledermatology to face-to-face consultation. It was consistently found that teledermatology yielded comparable outcomes to in-person appointments for the management of acne vulgaris.^33-38^

Several different scales and markers can be used to measure acne severity and characteristics. One study compared the reliability of the scores of IGA, Leeds Grading System, Total Inflammatory Lesion Counts (TILC), and Frontal Inflammatory Lesion Counts (FILC) across patients with acne both in-person and virtually. It was found that the reliability was highest for TILC and FILC scoring and lowest for IGA and Leeds.^39^ However, a reliability pilot study found that lesion count and IGA scores were agreeable between patient-taken digital photographs and in-person appointments.^36^ An additional study found that at the beginning of treatment, there was no significant difference in acne severity scores between the teledermatology and in-person groups [*P* = .18 for Global Acne Severity (GAS) scores, *P* = .67 for total lesion count (TLC)]. At the end of the study, it was found that changes in GAS and TLC scores were greater for the teledermatology group, but not significantly different.^39^ However, similarity in prescription patterns between in-person and virtual consultations is not guaranteed. Remote consultations, due to their novelty and reliance on patient-provided images, may tend toward more conservative approaches in prescribing medications. Consequently, dermatologists might prioritize monitoring and evaluating the efficacy of topicals before resorting to systemic medications. A 2022 study exemplified this trend, revealing a higher frequency of virtual visits resulted in topical prescriptions than systemic ones (72.5% vs 27.5% of the total, respectively).^40^ While adopting a conservative approach can be cost-effective if such methods prove effective for a patient, it may also result in a lengthier progression through various treatments before systemic intervention becomes necessary. This is particularly important for patients adversely impacted by delays, such as those with irreversible scarring who need prompt treatment. This nuanced dynamic can impact overall cost-effectiveness, potentially leading to increased health care costs for the patient over time.^41^ One solution for this, however, may be for dermatologists to include their first visit as an in-person appointment to accurately assess the lesion and decide on the best form of treatment, and move to virtual appointments for follow-up appointments and thus subsequent management. Although there are mixed conclusions, the results generally indicate that teledermatology and face-to-face appointments both achieve favourable and similar therapeutic outcomes for acne treatment.

### Patient Outcomes and Satisfaction with Teledermatology Interventions

While teledermatology appears to be comparable to in-person care for the management of acne, it is essential to consider patient satisfaction and experience. Three studies specifically focused on patient opinions of teledermatology in the context of acne management. The opinions of patients were favourable, with the finding that 100% of patients felt that their acne-related concerns were adequately addressed,^40^ and 71.1% of patients reported they were pleased with their acne treatment.^42^ Moreover, it was found that 71.8% of patients would select virtual video appointments if they were to see a dermatologist in future.^43^ 65.2% of patients on isotretinoin preferred video visits over in-person.^43^ The preference for teledermatology for patients on isotretinoin may be related to the number of additional tasks to be completed prior to receiving a prescription, specifically for females, including pregnancy tests, and in the United States, satisfying iPLEDGE requirements.^43^ Overall, patients were typically satisfied with the care provided via teledermatology.^40,43,44^

When comparing compliance between in-person appointments and teledermatology, it was found that teledermatology patients were less likely to follow up in the first 90 days than patients seen in-person (13% vs 31%).^45^ Additionally, patients seen virtually were significantly (*P* < .001) more likely to be treated with oral spironolactone (18.5% vs 12.5%) or antibiotics (43.0% vs 28.5).^45^ While it may be postulated that the younger patients are more likely to appreciate teledermatology due to their intrinsic tech-savviness from growing up in a virtual society, 1 study found the youngest age group, of under 17, was likely to drop out of teledermatology trials due to noncompliance when compared to patients seen in-person.^46^ However, this trend was less pronounced for the age groups of 17 to 21, and over 21.^46^ As such, the link between age and preference for teledermatology still requires more exploration.

### Potential Benefits and Challenges of Implementing Teledermatology in Acne Management

Although the treatment and management of acne vulgaris is the primary objective of teledermatology, there are several secondary benefits because of virtual care. Many patients found that virtual care eliminates the need to travel to and from appointments, with 94.8% of patients reporting such time savings.^46^ This reduction in time commitment could potentially increase patient adherence and outcomes.^40^ The reduction in time commitment that teledermatology offers is especially valuable in rural and remote areas, where patients may have to travel long distances regularly to see a dermatologist. A book by the Canadian Institute for Health Information on the geographic distribution of physicians in Canada reported that in a metropolitan area, with a population between 500,000 and 1,000,000 people, the average distance to a dermatologist is 6 km, while in a rural area with a population under 25,000, the average distance is 185.1 km.^47^ Not only is teledermatology of particular value in rural areas, where there is a 30-fold increase in travel distance required to see a dermatologist, but wait times have been demonstrated to be significantly longer (*P* = .002).^48^ With the implementation of teledermatology, it was reported in Nova Scotia that wait times for nonurgent dermatology appointments, such as for acne, was 4.6 weeks, compared with 13.7 weeks for a face-to-face consult.^49^ This difference in wait times and earlier intervention can significantly improve patient outcomes and reduce the occurrence of more significant adverse effects such as squalene of acne. Beyond travel and wait time, teledermatology in rural areas may make treatment with isotretinoin more accessible for patients.^34^ However, it is imperative to recognize that while teledermatology may mitigate barriers to access in rural areas, patients may still need to travel to a pharmacy to obtain prescription medications. Additionally, teledermatology is a promising way for primary care physicians to manage acne patients without the need to refer to a dermatologist.^50^

While there are several advantages to teledermatology for acne management, there are also limitations for both patients and physicians. The most common issues for patients utilizing virtual care included limited skin evaluations and trouble accessing virtual platforms.^40^ Additionally, some patients felt as though the dermatologist were able to see the texture of the skin, scars, and remaining acne better in-person than virtually.^40^ The quality of patient photographs may not be high enough or extend to areas such as the trunk, or patients may have limited range of shoulder and/or arm motion and be unable to capture photographs of the trunk region, which can delay access to care and commencement of treatment possibly resulting in worse outcomes. This is important for patients with irreversible scarring and other complex patients who may be impacted the most by these delays. As mentioned previously, one solution to this issue may be to have a first appointment in-person to best assess the lesion and then subsequent follow-up appointments virtually. In teledermatology, patients are less able to receive samples of over-the-counter products that may be used as part of one’s acne skin care routine. Studies used in this review focused on populations that had access to a mobile device, a camera, stable internet, and technological knowledge, which is not the case for all individuals and may pose a barrier to care. Teledermatology also presented unique challenges for physicians. One study detailed how the administrative process after a virtual appointment was considerably longer than at an in-person clinic,^51^ suggesting that a physician may not be able to see as many patients in a day when using teledermatology. Despite potential drawbacks for both patients and physicians, telemedicine is likely to persist postpandemic and there is a need to explore strategies to improve proficiency using the platform.^52 53^

### Future Directions in Teledermatology for Acne Management

Across the included studies, different teledermatology platforms were used, including but not limited to synchronous virtual calls, teledermatology portals, and asynchronous mobile phone photographs. Asynchronous teledermatology visits are more commonly used for acne, while synchronous teledermatology is likely more appropriate for medical dermatology with greater complexity.^52 53^ Several considerations, including the quality and accuracy of photographs, user compatibility of online systems, and equitable access for patients need to be made before widespread acceptance and implementation. Future studies are warranted to compare the effectiveness of telemedicine in both urban and rural areas as well as patient and physician opinions of each option to determine the most feasible way to implement teledermatology on a wider scale.

The synthesis of data from 21 studies on teledermatology’s application in acne vulgaris management consistently demonstrates its efficacy through reductions in acne scores comparable to that of in-person care. Consequently, in terms of favourable therapeutic outcomes, these 2 modalities are quantitatively equivalent. Patient opinions and satisfaction were overwhelmingly positive, emphasizing the desirability of teledermatology, and suggesting that it may be preferred over traditional face-to-face appointments. The differences observed in compliance rates and the writing of prescriptions for spironolactone or antibiotics highlight the nuanced considerations that need to be investigated when implementing teledermatology on a widespread scale. Overall, the findings of this study emphasize the feasibility of optimizing outcomes through teledermatology, while increasing accessibility and flexibility in the care and management of acne vulgaris.

## Conclusion

In conclusion, teledermatology has proven to be a promising tool for acne management, offering clinical effectiveness, time efficiency, and high patient satisfaction. Our review highlights its feasibility and effectiveness in managing acne vulgaris. However, challenges such as lower follow-up rates and variations in diagnostic accuracy necessitate further investigation. As teledermatology continues to gain prominence, future research should focus on exploring the efficiency and satisfaction from the use of various teledermatology platforms including perspectives from the most complex patients such as those with irreversible scarring, telemonitoring of treatment efficacy, and assessing long-term outcomes of teledermatology. Standardization of protocols and addressing accessibility challenges are critical for a successful permanent integration of telemedicine in dermatological practices.

## Supplemental Material

sj-docx-1-cms-10.1177_12034754241291028 – Supplemental material for Teledermatology and Virtual Visits for Acne Management: A Review Supplemental material, sj-docx-1-cms-10.1177_12034754241291028 for Teledermatology and Virtual Visits for Acne Management: A Review by Shanti Mehta, Dea Metko, Mahan Maazi, Ou Jia (Emilie) Wang and Monica K. Li in Journal of Cutaneous Medicine and Surgery

sj-docx-2-cms-10.1177_12034754241291028 – Supplemental material for Teledermatology and Virtual Visits for Acne Management: A Review Supplemental material, sj-docx-2-cms-10.1177_12034754241291028 for Teledermatology and Virtual Visits for Acne Management: A Review by Shanti Mehta, Dea Metko, Mahan Maazi, Ou Jia (Emilie) Wang and Monica K. Li in Journal of Cutaneous Medicine and Surgery

sj-docx-3-cms-10.1177_12034754241291028 – Supplemental material for Teledermatology and Virtual Visits for Acne Management: A Review Supplemental material, sj-docx-3-cms-10.1177_12034754241291028 for Teledermatology and Virtual Visits for Acne Management: A Review by Shanti Mehta, Dea Metko, Mahan Maazi, Ou Jia (Emilie) Wang and Monica K. Li in Journal of Cutaneous Medicine and Surgery
