# Supplementary material for: Teledermatology and Virtual Visits for Acne Management: A Review
Source: J Cutan Med Surg. 2024 Nov 1;29(1):63–8. doi: 10.1177/12034754241291028 (PMC11829499; doi:10.1177/12034754241291028)
Supplement: sj-docx-1-cms-10.1177_12034754241291028 – Supplemental material for Teledermatology and Virtual Visits for Acne Management: A Review [file sj-docx-1-cms-10.1177_12034754241291028.docx]

**SUPPLEMENTAL FILE 1**

**Appendix 1** Search terms (Search November 21st, 2023)

EMBASE

Keyword: Acne-related Terms: "acne fulminans", "acne conglobata", "acne keloidalis", "acne".mp, "acne cystica", "acne vulgaris", "acne", "acne excoriee"

Telemedicine and Digital Health-related Terms:"computer assisted therapy", "e consult*".mp, "econsult*".mp, "electronic consult*".mp, "e health".mp, "ehealth".mp, "e visit*".mp, "evisit*".mp, "home video visit*".mp, "internet", "web-based intervention", "internet".mp, "offsite care".mp, "off site care".mp, "telemedicine network".mp, "remote consultation*".mp, "remote visit*".mp, "tele care".mp, "telecare".mp, "teleconsultation", "tele consult*".mp, "teleconsult*".mp, "tele diagnos*".mp, "telehealth".mp, "tele health".mp, "telemedicine", "telemedicine".mp, "tele medicine".mp, "telemonitor*".mp, "tele monitor*".mp, "Telepathology", "telepatholog*".mp, "tele patholog*".mp, "telepractice*".mp, "tele practice*".mp, "video consult*".mp, "videoconsult*".mp, "virtual care".mp, "web based".mp, "teledermatology", "tele dermatolog*".mp, "teledermatolog*".mp

MEDLINE

Keyword: Acne-related terms: "acne fulminans", "acne conglobata", "acne keloidalis", "acne cystica", "acne vulgaris", "acne", "acne excoriee"

Telemedicine and digital health-related terms:"computer assisted therapy", "e consult*", "econsult*", "electronic consult*", "e health", "ehealth", "e visit*", "evisit*", "home video visit*", "internet", "web-based intervention", "offsite care", "off site care", "telemedicine network", "remote consultation*", "remote visit*", "tele care", "telecare", "teleconsultation", "tele consult*", "teleconsult*", "tele diagnos*", "telehealth", "tele health", "telemedicine", "tele monitor*", "Telepathology", "telepatholog*", "tele patholog*", "telepractice*", "tele practice*", "video consult*", "videoconsult*", "virtual care", "web based", "teledermatology", "tele dermatolog*", "teledermatolog*"

PubMed

(("acne fulminans", "acne conglobata", "acne keloidalis", "acne", "acne cystica", "acne vulgaris", "acne excoriee") AND ( "computer assisted therapy", "e consult*", "econsult*", "electronic consult*", "e health", "ehealth", "e visit*", "evisit*", "home video visit*", "internet", "web-based intervention", "offsite care", "off site care", "telemedicine network", "remote consultation*", "remote visit*", "tele care", "telecare", "teleconsultation", "tele consult*", "teleconsult*", "tele diagnos*", "telehealth", "tele health", "telemedicine", "tele medicine", "telemonitor*", "tele monitor*", "Telepathology", "telepatholog*", "tele patholog*", "telepractice*", "tele practice*", "video consult*", "videoconsult*", "virtual care", "web based", "teledermatology", "tele dermatolog*", "teledermatolog*"))
