# Supplementary material for: Teledermatology and Virtual Visits for Acne Management: A Review
Source: J Cutan Med Surg. 2024 Nov 1;29(1):63–8. doi: 10.1177/12034754241291028 (PMC11829499; doi:10.1177/12034754241291028)
Supplement: sj-docx-2-cms-10.1177_12034754241291028 – Supplemental material for Teledermatology and Virtual Visits for Acne Management: A Review [file sj-docx-2-cms-10.1177_12034754241291028.docx]

**Figure. S1**

**Identification of studies via databases and registers**

**Identification**

Records removed before screening:

Duplicate records removed

(n = 393)

Records identified from databases/registers

(n = 1499)

Medline = 211

Embase = 561

PubMed = 727

Records screened

(n = 1103)

Records excluded

Failed to meet title/abstract screening inclusion criteria

(n = 1014)

**Screening**

Reports not retrieved

(n =0)

Reports sought for retrieval

(n = 89)

Reports sought for retrieval

(n = 89)

Studies excluded (n = 68):

Wrong study design (n = 68)

**Included**

Studies included in review

(n = 21)
