# Supplementary material for: Teledermatology and Virtual Visits for Acne Management: A Review
Source: J Cutan Med Surg. 2024 Nov 1;29(1):63–8. doi: 10.1177/12034754241291028 (PMC11829499; doi:10.1177/12034754241291028)
Supplement: sj-docx-3-cms-10.1177_12034754241291028 – Supplemental material for Teledermatology and Virtual Visits for Acne Management: A Review [file sj-docx-3-cms-10.1177_12034754241291028.docx]

Table S1

| **Author, Year** | **Study type** | **Objective** | **n** | **Acne scale** | **Treatment** | **TD system** | **Versus in person** | **Study summary** |
| --- | --- | --- | --- | --- | --- | --- | --- | --- |
| Heidemeyer, 2023 | Randomized controlled trial | To compare teledermatology versus face-to-face consultation in the follow-up of patients with mild-to-moderate acne | 24 | IGA | NA | Teledermatology system (Evita®) as a portal for online advice service, and to use the related app for all acne assessments and follow-up | Similar outcomes in treatment and satisfaction | Acne is an optimal disease to follow using TD |
| Onyekweli, 2023 | Randomized controlled trial | To compare oral isotretinoin outcomes via asynchronous, synchronous, and hybrid teledermatology compared with in-person visits | 32 | NA | Isotretinoin | Not specified | All groups yielded similarly favorable therapeutic responses and adverse effect rate | TD may make treatment with isotretinoin more accessible for patients |
| Jusuf, 2023 | Cross sectional | To compare the severity of acne vulgaris by teledermatology with face-to-face consultations | 105 | International Consensus Conference on Acne Classification System | NA | Consultant based on photo documentation. | There are differences in the assessment of mild and severe acne vulgaris through teledermatology and face-to-face consultations due to factor such as the quality of images | The capability between TD and face-to-face consultations showed good conformity in assessing severity of acne vulgaris and is recommended in establishing severity of acne vulgaris if face-to-face consultation cannot be done |
| Moreno Ramirez, 2022 | Cross sectional | To assess the feasibility of store-and-forward teledermatology to manage patients with acne suitable for treatment with oral isotretinoin | 53 | GAGS/IGA | Isotretinoin | Mobile phone photos | NA | TD is feasible for the management of patients with moderate-to-severe acne who need oral isotretinoin |
| Gu, 2022 | Cross sectional | To analyze characteristics of acne visits during the pandemic | 505 | NA | Isotretinoin, spironolactone | Not specified | NA | About half of acne visits and most of the systemic acne management were conducted virtually even when in‐person visits were permitted |
| Giavina Bianchi, 2022 | Cross sectional | To assess how many referrals to in-person consultations with dermatologists could be avoided using asynchronous teledermatology in primary care attention | 2,459 | NA | NA | Mobile phone photos | NA | Asynchronous TD can be helpful to manage acne patients in primary care settings |
| Villani, 2022 | Cross sectional | To report the long‐term results of telemedicine visits for patients attending our outpatient clinic for acne disease | 213 | NA | Topical and systemic therapies | Mobile phone photos + video call | Half of patients (48.3%) decided to continue with telemedicine follow‐up visits, whereas 51.7% preferred in‐person visits | 48.3% of acne patients included preferred telemedicine even when in‐person visits were permitted |
| Hekman, 2021 | Survey | To evaluate patients’ perspectives of teledermatology for their isotretinoin visits | 46 | NA | Isotretinoin | Not specified | NA | 78.9% stated they would participate in video visits again under normal circumstances, and the majority (71.8%) responded that they would select video visits if they were to see a dermatologist in the future. For isotretinoin-related video visits, 65.2% of patients prefer video visits over in-person visits |
| Kazi, 2021 | Case control study | To provide quantitative data about the use of teledermatology | 2,823 | NA | NA | Real-time audio and/or video teleconferencing | NA | TD was effectively able to treat patients with acne remotely, and there was a greater number of diagnoses per visit with synchronous TD vs asynchronous TD |
| Khosravi, 2020 | Cohort study | To compare the rate and duration of follow-up between acne patients initially evaluated by teledermatology versus in-person outpatient consultation | 400 | NA | Topical, antibiotics, spironolactone | Not specified | NA | TD patients were less likely to follow-up in the first 90 days (13.0% versus 31.0%, P<0.001) compared to patients seen face-to-face with overall follow-up rates of 22% among both modalities and were more likely to be treated with oral antibiotics (43.0% versus 28.5%) or oral spironolactone (18.5% versus 12.5%) compared to patients seen face-to-face (P<0.001) |
| Ruggiero, 2020 | Observational prospective study | To assess how teledermatology visits were subjectively experienced by the patient as well as to identify how to improve the doctor–patient relationship and to satisfy patients’ expectations | 52 | NA | NA | Not specified | NA | 71% of patients were satisfied with the treatment they received, while 80.7% reported high well-being after treatments |
| Villani, 2020 | Cross sectional | To report our positive experience about the use of teledermatology for patients attending our outpatient clinic for acne disease | 72 | NA | Topical | Not specified | NA | All patients declared to be satisfied with this new web-procedure for TD |
| Singer, 2018 | Diagnostic test accuracy study (reliability pilot study) | To determine whether patient-taken photographs of acne using Network Oriented Research Assistant (NORA) result in similar lesion counts and Investigator’s Global Assessment (IGA) findings compared with in-person examination findings | 69 | NORA vs IGA | NA | Mobile phone photos | Strong agreement was observed between assessment scores from face-to-face examinations and those from patient-taken digital photographs | The Network Oriented Research Assistant can be used as a teledermatology platform for acne care |
| Fruhauf, 2015 | Randomized controlled trial | To investigate the superiority of mobile teledermatology in the care of patients with high-need facial acne in comparison to outpatient services with particular attention to treatment efficacy, safety, and patient compliance | 69 | GEA/TLC | Isotretinoin | Mobile phone photos | Results of the virtual were superior to those of the in person, however, no statistically significant difference could be observed between the groups | Mobile TD is an efficient, safe, and well-accepted tool among patients with high-need acne constituting at least a valuable adjunct to outpatient care services |
| Watson, 2009 | Randomized controlled trial | To evaluate whether delivering acne follow-up care via an asynchronous, remote online visit (evisit) platform produces equivalent clinical outcomes to office care | 151 | TILC, FILC, leeds | NA | Mobile phone photos | Acne improvement and satisfaction was similar in the e-visit and office visit groups | Total inflammatory lesion count was similar in the evisit and office visit groups (P=0.51). Both subjects and dermatologists reported comparable satisfaction with care regardless of modality (P=0.16) |
| Bergman, 2009 | Cross sectional | To determine whether specific assessment tools designed to grade acne during faceto-face visits can be applied to the evaluation of digital images | 20 | TILC, FILC, leeds, IGA | NA | Mobile phone photos | NA | Digital images of inflammatory acne lesions could reliably be evaluated using certain clinical assessment tools |
| Watson, 2021 | Diagnostic test accuracy study (pilot) | To examine the use of MySkinSelfie app | 11 | DLQI, PHQ9 | Isotretinoin | MySkinSelfie app | NA | It is possible to safely deliver a remote acne service using the MySkinSelfie app; however, there were several logistical challenges |
| Shah, 2022 | Cross sectional | To evaluate patients’ experiences and satisfaction with teledermatology particularly for patients on isotretinoin for acne management | 14 | NA | Isotretinoin | Mobile phone photos + video call | NA | Given dermatologists’ comfort managing acne virtually and positive patients’ experience with TD, supporting future coverage of virtual visits by iPLEDGE and insurances may reduce treatment burden on patients and ultimately increase treatment adherence |
| Das, 2022 | Retrospective study | To pilot a direct-care asynchronous telemedicine program for isotretinoin management | 143 | NA | Isotretinoin | Web portal | Dosing outcomes of isotretinoin were not different between AT and ST groups. | Dermatologists were comfortable remotely adjusting isotretinoin dosing (both escalating for therapeutic effect and decreasing to manage side effects) without a synchronous encounter |
| Munoz, 2022 | Cross sectional | To investigate and compare patient satisfaction with recorded video counseling versus traditional, in-office counseling | 16 | NA | Isotretinoin | Not specified | There was similar satisfaction between in person and virtual care. | The study was conducted to evaluate patient satisfaction with video counseling compared to traditional in-office counseling for isotretinoin therapy, a treatment for severe acne. The study randomized patients into two groups: one receiving video counseling (11 patients) and another receiving face-to-face counseling (5 patients). They then completed a survey on various satisfaction and comfort aspects. The findings revealed no significant difference in satisfaction levels between the two methods, indicating that video counseling is an effective alternative |
| Duarte, 2022 | Prospective longitudinal study | To assess the feasibility of managing and controlling moderate to severe acne with isotretinoin through teledermatology | 46 | GAGS | Isotretinoin | Mobile phone photos | NA | TD has been suitable for the diagnosis and treatment of patients with moderate-to-severe acne with oral isotretinoin. |

Legend: TD, teledermatology; IGA, Investigator's Global Assessment; PHQ9, Patient Health Questionnaire 9; DLQI, Dermatology Life Quality Index; GAGS, Global Acne Grading System; GES, Global Acne Severity Scale; TLC, Total Lesion Counting Scale; NORA, Network Oriented Research Assistant; TILC, Total Inflammatory Lesion Counts; FILC, Frontal Inflammatory Lesion Counts
